# Asymptomatic Choledocholithiasis that Causes a Dilemma between Treatment and Observation

**DOI:** 10.31662/jmaj.2021-0025

**Published:** 2021-04-02

**Authors:** Masaki Kuwatani, Naoya Sakamoto

**Affiliations:** 1 Division of Endoscopy, Hokkaido University Hospital, Sapporo, Japan; 2 Department of Gastroenterology and Hepatology, Hokkaido University Hospital, Sapporo, Japan

**Keywords:** choledocholithiasis, common bile duct stone, endoscopic retrograde cholangiopancreatography, pancreatitis

It has long been believed that choledocholithiasis (common bile duct stone), whether symptomatic or asymptomatic, should be treated considering the risk of jaundice, acute cholangitis, or acute pancreatitis. There is almost no debate among clinicians about treating symptomatic choledocholithiasis, which can occasionally lead to life-threatening conditions. However, managing asymptomatic choledocholithiasis is becoming controversial. Any treatment carries a certain frequency of risk of adverse events, and endoscopic treatment with endoscopic retrograde cholangiopancreatography (ERCP) is no exception. Kadokura et al. indicated the increased risk of post-ERCP pancreatitis in patients with asymptomatic choledocholithiasis and also raised the issue whether it should be treated or not ^(1)^. There have been eight relevant reports and four national guidelines from the National Institute for Health and Care Excellence in the United Kingdom (NICE), European Society of Gastrointestinal Endoscopy (ESGE), British Society of Gastroenterology (BSG), and European Association for the Study of the Liver (EASL) on managing asymptomatic choledocholithiasis ^(2)^. Among those, four reports included the natural history of asymptomatic choledocholithiasis, three of which also showed feasibility of the wait-and-see strategy because of spontaneous stone clearance (22/114 and 12/34 patients) ^(3), (4)^, no biliary complication (14/14 patients) during observation ^(5)^ or the higher risk of ERCP-related complications (32%) compared to the risk of biliary events during the wait-and-see period (6.1% at 1 year, 11% at 3 years, and 17% at 5 years) ^(3)^. Meanwhile, three of the four national guidelines (NICE, ESGE, and BSG) support clearance of asymptomatic common bile duct stone by some kind of procedure even though there is little data on natural history of asymptomatic choledocholithiasis and no randomized controlled trial between intervention and observation. As a result, we are now in a dilemma.

In order to get out of the dilemma, we need to know two important facts about asymptomatic choledocholithiasis: 1) accurate natural history with a large cohort (maybe by each country, or by stone size) and 2) predictors, risk factors, and frequencies of intervention-related adverse events, especially post-ERCP pancreatitis. First, we require prospective and largescale cohort studies by a national or official academic society. Second, three of the eight studies mentioned above have given us the clues ^(6), (7), (8)^. The collective results indicated that a higher incidence of post-ERCP pancreatitis in the asymptomatic group (12.5-20.8%) than in the symptomatic group (3-6.9%) would be attributed to nondilated CBD, with small ampullary orifice, and prolonged cannulation time in the asymptomatic group, although the study by Kadokura et al. ^(1)^ couldn’t reveal the reason for that. Therefore, a well-designed randomized controlled trial or meta-analysis with a multivariate analysis to identify predictors and risk factors for intervention-related adverse events is warranted.

Apart from the treatment strategy against asymptomatic choledocholithiasis, the accurate diagnosis of choledocholithiasis is also extremely important. The American Society of Gastrointestinal Endoscopy guidelines, published in 2019, suggest either endoscopic ultrasonography (EUS) or magnetic resonance cholangiopancreatography (MRCP) to confirm the diagnosis according to factors such as patient preference, local expertise, and resource availability (conditional recommendation, low quality of evidence) ^(9)^, although Kadokura et al. ^(1)^ selected MRCP for the diagnosis. EUS by skillful experts yields highest sensitivity (EUS, 0.97 [95% confidence interval [CI], .91-.99] vs MRCP, 0.87 [95% CI, .80-.93], P = .006). and diagnostic accuracy (Odds ratio was greater for EUS (162.5 [95% CI, 54.0-489.3]) than MRCP (79.0 [95% CI, 23.8-262.2], P = .008)) for choledocholithiasis among various imaging modalities ^(9)^; however, EUS may not be feasible due to lack of expertise or equipment. Therefore, in such situations without EUS, clinicians should be most cautious in making the diagnosis, considering false positives and negatives.

Although we cannot conclude the pros and cons of ERCP-guided treatment of asymptomatic choledocholithiasis at present, we have to consider its indication according to each patient’s condition and discuss the strategy with each patient, sincerely confronting the dilemma between risks and benefits of the treatment.

## Article Information

### Conflicts of Interest

None

### Disclaimer

Naoya Sakamoto is one of the Editors of JMA Journal and on the journal’s Editorial Staff. He was not involved in the editorial evaluation or decision to accept this article for publication at all.
